# Malignancy Risk and Related Factors of Atypia of Undetermined Significance/Follicular Lesion of Undetermined Significance in Thyroid Fine Needle Aspiration

**DOI:** 10.1155/2018/4521984

**Published:** 2018-07-30

**Authors:** So-hyeon Hong, Hyejin Lee, Min-Sun Cho, Jee Eun Lee, Yeon-Ah Sung, Young Sun Hong

**Affiliations:** ^1^Division of Endocrinology and Metabolism, Department of Internal Medicine, Korea University College of Medicine, Seoul, Republic of Korea; ^2^Division of Endocrinology and Metabolism, Department of Internal Medicine, College of Medicine, Ewha Womans University, Seoul, Republic of Korea; ^3^Department of Pathology, College of Medicine, Ewha Womans University, Seoul, Republic of Korea; ^4^Department of Radiology, College of Medicine, Ewha Womans University, Seoul, Republic of Korea

## Abstract

Atypia of undetermined significance/follicular lesion of undetermined significance (AUS/FLUS) in thyroid fine needle aspiration (FNA) is a challenging category. The malignancy risk is different by multiple factors and subsequent management strategy is inconclusive. Therefore, we analyzed the malignancy risk of AUS/FLUS according to radiological and clinical features. A total of 687 nodules that had been initially diagnosed as AUS/FLUS were retrospectively reviewed from 6365 thyroid FNAs between 2011 and 2014. The ultrasonographic (US) features were categorized using the Korean Thyroid Imaging Reporting and Data System. Radiological and clinical features were compared according to the second FNA results or histologically confirmed results from surgery. Repeat FNA was performed on 248 (36%) nodules, and 49 (7%) nodules underwent immediate surgery. Among the 248 nodules subjected to repeated FNA, 49 (20%) nodules were diagnosed again as AUS/FLUS, 123 (50%) were found to be benign, and 47 (19%) were diagnosed as follicular neoplasm, suspicious for malignancy or malignant. Among histologically confirmed nodules, the US features were more unfavorable in malignant nodules, and hypo- or anechogenicity was associated with a higher risk of malignancy after adjusting for age, size, and other US features (*P* < 0.01). In conclusion, we observed that malignant nodules tended to show unfavorable US features, especially hypo- or anechogenicity. Age, sex, and thyroid function were not significantly associated with malignancy risk. We also found out that malignancy risk was not different between the group which underwent immediate operation following the AUS/FLUS diagnosis and the group which underwent repeated FNA after the initial diagnosis.

## 1. Introduction

Thyroid nodules are commonly observed, and the prevalence of incidental discovery by ultrasound (US) or other radiologic studies is reported to be in the wide range of 20–76% [1–3]. Fine needle aspiration (FNA) is recommended for clinically indicated thyroid nodules, and the result is reported according to 6 Bethesda categories. Among them, Bethesda category III, atypia of undetermined significance/follicular lesion of undetermined significance (AUS/FLUS), accounts for 7–18% of the thyroid nodules [4–6]. The category is a heterogeneous group that consists prominently of microfollicles or focal nuclear atypia but otherwise does not fulfill the criteria of follicular neoplasm or papillary carcinoma [4]. The 2015 American Thyroid Association Management Guidelines recommend a repeat FNA or molecular test to supplement the assessment of malignancy risk and to decide whether active surveillance or diagnostic surgery is needed [7]. However, the accuracy in diagnosis, such as benign or malignant, after the repeated FNA in AUS/FLUS is only about 50% [8], and supplementary methods such as molecular tests and ultrasonography have shown only limited efficacy [9, 10].

The malignancy rate for AUS/FLUS has been estimated to be between 5 and 15% [4]. However, recent studies reported values in the range of 6% to 48% [4, 11–16]. One study reported the malignancy rate of AUS as 6.3% for all cases and 26.1% for surgical follow-up cases [14], and another study reported the malignancy rate of AUS/FLUS to be 26.6% for total cases and 37.8% for those that underwent excision [5]. The meta-analyses estimated the malignancy rate to be in the range of 15.9% to 37.8% [13, 16].

Repeated FNA for definite category assignment of AUS/FLUS is also controversial. One study reported that the malignancy rate of direct surgical diagnosis after the first AUS/FLUS and repeated AUS/FLUS diagnoses were statistically not different [6]. Some studies demonstrated the malignancy rate of AUS/FLUS using US and/or pathologic subcategories, but validation was unsuccessful in lower cancer risk populations [17, 18].

Herein, we report the malignancy rate of the AUS/FLUS category in thyroid nodules from our institution's four-year experience. We also compared the radiological and clinical factors according to second FNA results and/or final histologically confirmed results to identify and evaluate malignancy-related features.

## 2. Materials and Methods

### 2.1. Patients and FNA Specimen

A total of 6365 thyroid nodules, which underwent US-guided FNA at the Ewha Womans University Mokdong Hospital from January 2011 to December 2014, were retrospectively reviewed. Among the nodules, 717 from 687 patients were classified as Bethesda class III and included in our analysis. In the case of patients with multiple nodules which underwent FNAs, one was chosen based on larger size or more unfavorable US features. We compared the basal characteristics among the groups with repeat FNA, thyroidectomy, and follow-up loss after being first diagnosed as AUS/FLUS to identify and minimize the selection bias.

All nodules underwent thyroid US using 5–12 MHz (iU22; Philips Medical System, Bothell, WA), 4–15 MHz (Aixplorer; Supersonic Imagine, Aix en Provence, France), and 6–15 MHz (LOGIQ E9; GE Medical Systems, Milwaukee, WI, USA) linear transducers. US-guided FNA was done by board-certified radiologists using a 24-gauge needle with typically one or two passes. The slides were alcohol-fixed for hematoxylin and eosin and Papanicolaou staining. The slides were prepared using the ThinPrep 2000 (Hologic, Marlborough, MA).

### 2.2. US Examination

The US features were categorized using the Korean Thyroid Imaging Reporting and Data System (K-TIRADS) which defines US characteristics as low suspicion (K-TIRADS 3), intermediate suspicion (K-TIRADS 4), and high suspicion (K-TIRADS 5) categories [18]. K-TIRADS 3 nodules are those with partially cystic or iso/hyperechoic features without any of the 3 suspicious US features. K-TIRADS 4 nodules are solid hypoechoic nodules without any of the 3 suspicious US features or partially cystic or isoechoic nodules with any of 3 suspicious US features. K-TIRADS 5 nodules are solid hypoechoic nodules with any of the 3 suspicious US features. Suspicious US features include microcalcification, nonparallel orientation (i.e., taller-than-wide), and speculated/microlobulated margin [18]. Tumor size was measured as the maximum diameter of the gland.

All thyroid nodules were described according to the following categories: size, echogenicity, shape, calcification, composition, margin, and orientation. The size was measured as a maximal diameter, and echogenicity was classified as hyperechogenicity, isoechogenicity, hypoechogenicity, or anechogenicity. The shape was classified as round to oval or irregular, and the composition was classified as solid, predominantly solid, predominantly cystic, and cystic. The margin was classified as smooth or irregular, and the orientation was classified as parallel or nonparallel. We could not analyze the composition, margin, and orientation for the analysis due to an absence of cystic or predominantly cystic nodules in all histologically confirmed nodules and an absence of smooth margin and nonparallel nodules in histologically confirmed benign nodules.

### 2.3. Cytologic and Histologic Diagnosis

FNA specimens were reviewed by experienced pathologists following the Bethesda System for Reporting Thyroid Cytopathology [4]. Bethesda category III—AUS/FLUS—diagnosis is made if a cytology specimen contains cells having architectural and/or nuclear atypia in the case of AUS or contains a predominantly microfollicular pattern but sparse cellularity with scanty or no colloid or predominance of oncocytic cells but sparse cellularity in the case of FLUS [4].

### 2.4. Statistical Analysis

The statistical analysis was performed using the SPSS 20.0 software package for Windows (IBM Corp., Chicago, IL, USA). Comparison of two groups with quantitative variables was analyzed using Student's *t*-test, and the three groups were analyzed using ANOVA. Categorical variables were performed by chi-square analysis. Analysis of the three groups was done using ANOVA. A multiple logistic regression analysis was performed to identify the variables predicting malignant nodules. All *P* values of <0.05 were considered to have statistical significance.

## 3. Results

**Figure 1:**
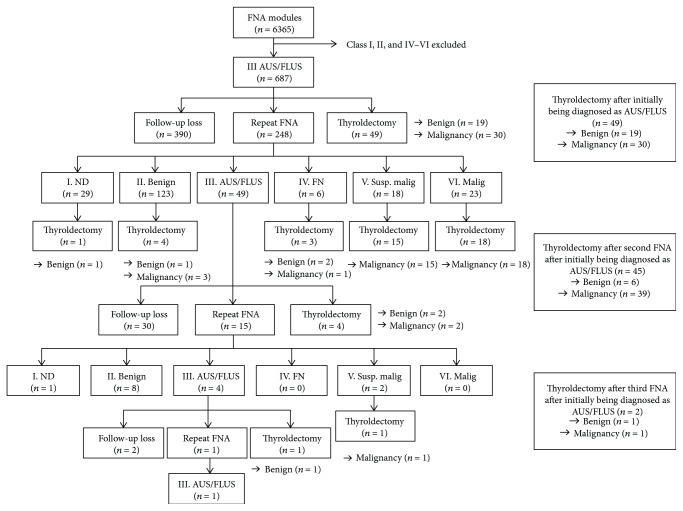
Flow diagram of the clinical course of AUS/FLUS nodule patients. *n*: the number of thyroid nodules; AUS/FLUS: atypia of undetermined significance/follicular lesion of undetermined significance; FNA: fine needle aspiration; ND: nondiagnostic; FN: follicular neoplasm; Susp. malig: suspicious for malignancy; Malig: malignant.

The clinical course of patients initially diagnosed as AUS/FLUS is presented in Figure 1. In a total of 687 thyroid nodules initially diagnosed as AUS/FLUS, 7% (*n* = 49) underwent thyroidectomy, which provided a histologic diagnosis. 61% (*n* = 30) of those nodules were malignant, and 39% (*n* = 19) were benign. Another 248 nodules were subjected to repeated FNA. The results of the second FNA on the nodules were categorized as follows: 20% (*n* = 49) in AUS/FLUS, 50% (*n* = 123) in benign, 19% (*n* = 47) in follicular neoplasm, suspicious malignant, or malignant, and 11% (*n* = 29) in nondiagnostic categories. Since then, a total of 45 nodules, 4 in AUS/FLUS, 4 in benign, 36 in follicular neoplasm, suspicious malignant, and malignant, and 1 in nondiagnostic categories, underwent surgery, and 39 cases were finally diagnosed as malignant. In the first and second diagnoses of AUS/FLUS nodules, 15 nodules underwent a third FNA. 27% (*n* = 4) of those nodules were diagnosed as AUS/FLUS for the third consecutive time, and 53% (*n* = 8) were diagnosed as benign, while 13% (*n* = 2) of the nodules were diagnosed as suspicious malignant, and 1 nodule was reported as a nondiagnostic category.

The basal characteristics of 687 thyroid nodules with three groups—repeat FNA, thyroidectomy, and follow-up loss after being first diagnosed as AUS/FLUS—are presented in Table 1. There were no differences in the three groups for age, sex, TSH and free T4 levels, and US findings with the exception of the nodule size. The nodules which underwent thyroidectomy had a larger average diameter than those which underwent repeated FNA (18.7 ± 16.8 mm versus 11.8 ± 7.8 mm, *P* < 0.01).

Table 2 represents the basal characteristics, according to the second FNA results, of the nodules subjected to repeat FNA after first being diagnosed as AUS/FLUS. Age, sex, and levels of TSH and free T4 were not different among them. Nodule size was significantly smaller in the malignant group than the benign and the AUS/FLUS group (7.9 ± 4.4 mm, 13.0 ± 8.5 mm, and 12.2 ± 7.4 mm, resp., *P* < 0.01). K-TIRADS score was higher in the malignancy group than the benign and the AUS/FLUS groups (4.2 ± 0.6, 3.3 ± 0.5, and 3.5 ± 0.6, resp., *P* < 0.01).

We compared the histologically confirmed malignancy rates between the group which underwent direct thyroidectomy after first being diagnosed as AUS/FLUS and the group which underwent thyroidectomy after two or more consecutive FNAs after first being diagnosed as AUS/FLUS. The group of thyroidectomy after repeated FNA showed higher malignancy rate compared to the group of direct thyroidectomy (61% versus 85%, *P* = 0.01, S1 Table).

Table 3 represents the basal characteristics of histologically confirmed benign and malignant nodules after initially being diagnosed as AUS/FLUS. Age, sex, and TSH and free T4 levels were not different between benign and malignant nodules. Nodule size was significantly smaller in malignant nodules compared to benign nodules (9.9 ± 11.3 mm and 24.9 ± 13.9 mm, resp., *P* < 0.01). K-TIRADS score was higher in malignant nodules compared to benign nodules (4.1 ± 0.7 and 3.2 ± 0.4, resp., *P* < 0.01).

Logistic regression analysis showed that the malignant nodule was significantly associated with hypo- or anechogenicity (OR = 9.31 (95% CI 1.89–45.9), *P* < 0.01) after adjustment for age, size, shape, and calcification (Table 4). We could not analyze the composition, margin, and orientation for analysis due to an absence of partially cystic or cystic nodules in all histologically confirmed nodules and an absence of smooth margin and nonparallel nodules in histologically confirmed benign nodules.

## 4. Discussion

In this study, we observed that malignant nodules in AUS/FLUS showed unfavorable US features compared to benign nodules in AUS/FLUS. Malignant nodules were associated with hypo- or anechogenicity in AUS/FLUS nodules. There was no difference in the malignancy rate between the groups which underwent direct surgery after being first diagnosed as AUS/FLUS and the group which underwent surgery following repeat FNA after being first diagnosed as AUS/FLUS.

The Bethesda System for Reporting Thyroid Cytopathology categorized thyroid nodules by 6 classes, estimated malignancy risk factor, and provided clinicians to make a useful decision [4]. The Bethesda category III, AUS/FLUS, represents a heterogeneous population, which is not clearly benign or malignant. It presents cellular architectural and/or nuclear atypia but not sufficient to diagnose malignancy or follicular neoplasm [4]. Although malignancy risk is regarded to be between 5% and 15%, recent studies report a range of 6% to 48% [11–15]. This broad range of malignancy risk is due to a difference in severity of study populations from primary to tertiary institutions and variant denominator inclusions across studies—for example, only histologically confirmed nodules, adding repeated FNA results, and/or follow-up loss. Therefore, a cautious approach is needed for AUS/FLUS nodules to establish an institution-specific malignancy risk and develop detailed subcategorization of the AUS/FLUS to predict accurate malignancy risks.

To calculate a thorough malignant risk of AUS/FLUS, all nodules need to be followed up and histologically diagnosed. These are nearly impossible in clinical settings and lead to selection bias. Several strategies were designed to resolve the bias. One study adjusted inclusion criteria so as to include not only surgical nodules confirmed histologically but also the nodules that had been diagnosed as benign or malignant upon follow-up FNA. Both stable nodules and those that showed decreased size after at least a 12-month follow-up were also included [19]. Other studies calculated upper and lower limits of malignancy risk, with the upper limit estimating malignancy rate in operated cases only and the lower limit calculating malignancy rate in all operated and nonoperated cases, assuming all of the nonoperated cases were benign [14, 15]. Applying this calculation, our institution's malignancy risk is 72.9% (*n* = 70/96) in the upper boundary limit and 10.2% (*n* = 70/687) in the lower boundary limit. Because of a significant follow-up loss in our study, estimating malignancy risk is in a broad range and it is difficult to lead a clinical decision.

Some studies subcategorized AUS based on cytomorphology. One study subclassified AUS into INa (low cellularity with predominant microfollicular architecture) and INb (absence of colloid and nuclear features not characteristic of benign lesions) and found out that malignancy risk was statistically higher in the INa group [20]. Another study divided AUS into four subgroups—AUS cannot exclude follicular neoplasm, AUS cannot exclude Hurthle cell neoplasm, AUS cannot exclude papillary carcinoma, and AUS not otherwise specified. The four different groups showed significantly different malignancy risks [21]. However, the attempts are subjective by pathologists and not yet validated by other studies.

US features of malignant thyroid nodules are well recognized. Korean Society of Thyroid Radiology recommended that in AUS/FLUS cytology, low to intermediate suspicion for malignancy in nodules (K-TIRADS 3 and K-TIRADS 4) should lead to repeated FNA within 6–12 months, and in the case of high suspicion for malignancy in nodules (K-TIRADS 5), FNA should be repeated within 3–6 months [18]. Our institution followed the K-TIRADS reporting system for evaluating US malignancy risk and it showed a higher score in malignant nodules. Taken together, the US features could provide a significant diagnostic value for predicting malignancy risk. Among the US features taken into account in K-TIRADS, hypo- or anechoic nodules were significantly associated with malignant nodules in our results. Subcategorization of US features showed different results among studies. One study reported that the taller-than-wide shape and microcalcification were associated with malignant nodule [22]. In contrast, another study demonstrated that taller-than-wide shape, ill-defined margin, and micro- or macrocalcification had higher odds of malignancy (compared with an oval shape, well-defined margin, and no calcification, resp.) [23]. We could not analyze the composition, margin, and orientation due to an absence of smooth margin and/or nonparallel nodules in histologically confirmed benign nodules.

It is interesting that, in our study, malignant nodules are markedly smaller than benign nodules in AUS/FLUS, in contrast to previous studies. One previous study reported that in all thyroid nodules, increased size impacted cancer risk in a nonlinear fashion, with the threshold size of 2.0 cm [24]. Another study reported that larger nodules showed a higher probability of malignancy, and nodules larger than 3 cm had reduced accuracy in FNA diagnosis [25]. We attribute our observation to the tendency of physicians to be reluctant to perform thyroidectomy of smaller nodules unless substantial evidence of malignancy is present. In contrast, thyroidectomy, rather than repeating FNA, is preferred on larger nodules. We believe that the size-dependent approach toward the nodules forms the basis of the apparent increased rate of malignancy in smaller nodules. However, considering that the K-TIRADS criteria do not account for nodule size and that size was not significantly related to the malignant nodule in logistic regression analysis, size may not be a critical factor in evaluating AUS/FLUS nodule.

Our study showed that the malignancy rates from the direct surgical diagnosis after the first AUS/FLUS and from the surgical diagnosis after repeating FNA were not different. These results are consistent with a previous study showing that the malignancy rates of patients who underwent thyroidectomy directly after the first AUS and those who had two successive AUS diagnoses were not different [6]. Thyroidectomy is more burdensome than repeat FNA in patients, and second AUS/FLUS can provide a conclusive diagnosis in some cases. So repeat FNA could be a reasonable choice in the nodules initially diagnosed with AUS/FLUS.

Outside the US features of thyroid nodules, recent studies reported that molecular mutation tests or US patterns of lymph nodes were helpful in evaluating their malignancy risk. The molecular mutation test and US patterns of lymph nodes even allowed for the prediction of the possibility of recurrence in papillary thyroid cancer [26–28]. Although we could not evaluate regional lymph nodes or conduct mutation test in this study, further studies on comprehensive components predicting malignant risk for AUS/FLUS are required.

The strength of our study is in the relatively large number of thyroid nodules and the consideration of a diverse clinical, US, and pathological assessment in determining malignancy risk of AUS/FLUS nodules. The limitation of the study is that there was a considerable follow-up loss, and molecular mutation tests were not applied for the additional diagnostic tools for predicting malignancy risk in AUS/FLUS.

## 5. Conclusions

We observed that the malignant nodule was related to unfavorable US features compared to benign nodules in the AUS/FLUS group. Notably, hypo- or anechogenicity was strongly associated with a malignancy risk in AUS/FLUS. The malignancy risks of the direct thyroidectomy after the first diagnosis of AUS/FLUS and of the thyroidectomy after repeated FNA were not different. Further studies predicting malignancy risk are needed to develop a proper treatment strategy for AUS/FLUS nodules.

## Figures and Tables

**Table 1:** Basal characteristics among groups with repeated FNA, thyroidectomy, and follow-up loss after initially being diagnosed as AUS/FLUS.

	Number of nodules	Age (yrs)	Number of women (%)	TSH (uIU/mL)	Free T4 (ng/dL)	Size (mm)	K-TIRADS
Repeated FNA	248	52 ± 11	216 (87.1)	2.47 ± 2.99	1.22 ± 0.22	11.8 ± 7.8	3.53 ± 0.62
Thyroidectomy	49	51 ± 11	43 (87.8)	2.20 ± 1.67	1.31 ± 0.34	18.7 ± 16.8	3.71 ± 0.76
Follow-up loss	390	53 ± 12	344 (88.2)	2.43 ± 4.26	1.26 ± 0.51	—	—
*P* value		0.54	0.92	0.90	0.49	<0.01	0.12

FNA: fine needle aspiration; AUS/FLUS: atypia of undetermined significance/follicular lesion of undetermined significance; K-TIRADS: Korean Thyroid Imaging Reporting and Data System.

**Table 2:** Basal characteristics of repeated FNA after initially being diagnosed as AUS/FLUS according to the second FNA results.

	Number of nodules	Age (yrs)	% of women	TSH (uIU/mL)	Free T4 (ng/dL)	Size (mm)	K-TIRADS
Repeated FNA							
II. Benign	123	52 ± 12	89.1	2.19 ± 1.74	1.23 ± 0.19	13.0 ± 8.5§	3.4 ± 0.5§
III. AUS/FLUS	49	52 ± 11	78.4	3.02 ± 5.56	1.20 ± 0.24	12.2 ± 7.4¶	3.5 ± 0.6¶
IV. FN	47	51 ± 10	89.8	2.62 ± 2.26	1.21 ± 0.25	7.9 ± 4.4§¶	4.2 ± 0.6§¶
V. Susp. malig							
VI. Malig							
*P* value		0.69	0.93	0.32	0.74	<0.01	<0.01

Data are the mean ± standard deviation. §: *P* value < 0.05 for II. Benign versus IV. FN, V. Susp. malig, and VI. Malig; ¶: *P* value < 0.05 for III. AUS/FLUS versus IV. FN, V. Susp. malig, and VI. Malig; FNA: fine needle aspiration; AUS/FLUS: atypia of undetermined significance/follicular lesion of undetermined significance; K-TIRADS: Korean Thyroid Imaging Reporting and Data System; FN: follicular neoplasm; Susp. malig: suspicious for malignancy; Malig: malignant.

**Table 3:** Comparison of characteristics between surgically confirmed (histologically confirmed?) benign and malignant nodules after initially being diagnosed as AUS/FLUS.

	Number of nodules	Age (yrs)	Number of women (%)	TSH (uIU/mL)	Free T4 (ng/dL)	Size (mm)	K-TIRADS
Benign	26	51 ± 11	23 (88.5)	2.18 ± 1.86	1.30 ± 0.38	24.9 ± 13.9	3.2 ± 0.4
Malignant	70	52 ± 11	64 (91.4)	2.42 ± 1.98	1.25 ± 0.22	9.9 ± 11.3	4.1 ± 0.6
*P* value		0.66	0.66	0.59	0.59	<0.01	<0.01

AUS/FLUS: atypia of undetermined significance/follicular lesion of undetermined significance; K-TIRADS: Korean Thyroid Imaging Reporting and Data System.

**Table 4:** Logistic regression analysis of predictors of malignancy in histologically confirmed thyroid nodules after initially being diagnosed as AUS/FLUS.

	Age-adjusted OR (95% CI)	*P* value	Multivariate-adjusted^∗^ OR (95% CI)	*P* value
Size	0.90 (0.86–0.95)	<0.01	0.96 (0.91–1.02)	0.20
Hyper- or isoechoic versus hypo- or anechoic	18.81 (5.89–60.04)	<0.01	9.31 (1.89–45.9)	<0.01
Round to oval versus irregular shape	2.42 (0.64–9.10)	0.19	0.79 (0.09–6.88)	0.87
No calcification versus calcification	0.76 (0.29–1.97)	0.58	0.56 (0.15–2.07)	0.38
Smooth versus irregular margin	0.00	0.99	0.00	0.99
Parallel versus nonparallel	783,560,819 (?)	0.99	197,355,593(?)	0.99

^∗^Adjusted for age, size (as a continuous variable), and all the other variables listed in the table. AUS/FLUS: atypia of undetermined significance/follicular lesion of undetermined significance.

## Data Availability

The data used to support the findings of this study are available from the corresponding author upon request.

## Supplementary Materials

Supplementary Materials S1 Table: histologic diagnosis between the groups of direct thyroidectomy and of thyroidectomy after two or more consecutive FNAs after initially being diagnosed as AUS/FLUS.
