# Supplementary material for: Malignancy Risk and Related Factors of Atypia of Undetermined Significance/Follicular Lesion of Undetermined Significance in Thyroid Fine Needle Aspiration
Source: Int J Endocrinol. 2018 Jul 30;2018:4521984. doi: 10.1155/2018/4521984 (PMC6091291; doi:10.1155/2018/4521984)
Supplement: Supplementary Materials — S1 Table: histologic diagnosis between the groups of direct thyroidectomy and of thyroidectomy after two or more consecutive FNAs after initially being diagnosed as AUS/FLUS. [file 4521984.f1.docx]

Supplement table 1. Histologic diagnosis between the groups of direct thyroidectomy and of thyroidectomy after two or more consecutive FNAs after initially being diagnosed as AUS/FLUS (*P* = 0.01)

| Histologic diagnosis | No. of nodules (%) | Direct thyroidectomy after first diagnosed as AUS/FLUS | Thyroidectomy after two or more consecutive FNAs after first diagnosed as AUS/FLUS |
| --- | --- | --- | --- |
|  |  | No. of nodules (%) | No. of nodules (%) |
| Benign | 26 (27) | 19 (39) | 7 (15) |
| Malignant | 70 (73) | 30 (61) | 40 (85) |
| Total | 96 (100) | 49 (100) | 47 (100) |

FNA, Fine needle aspiration; AUS/FLUS, Atypia of undetermined significance/follicular lesion of undetermined significance.
